# Perfusion Patterns of Peripheral Organizing Pneumonia (POP) Using Contrast-Enhanced Ultrasound (CEUS) and Their Correlation with Immunohistochemically Detected Vascularization Patterns

**DOI:** 10.3390/diagnostics11091601

**Published:** 2021-09-02

**Authors:** Ehsan Safai Zadeh, Christina Carolin Westhoff, Corinna Ulrike Keber, Corinna Trenker, Christoph Frank Dietrich, Amjad Alhyari, Charlotte Gabriele Luise Mohr, Christian Görg

**Affiliations:** 1 Interdisciplinary Center of Ultrasound Diagnostics, University Hospital Giessen and Marburg, Philipps University Marburg, Baldingerstraße, 35033 Marburg, Germany; ehsan_sz@yahoo.de (E.S.Z.); charlottemohr96@gmx.de (C.G.L.M.); 2 Institute of Pathology, University Hospital Giessen and Marburg, Philipps University Marburg, Baldingerstraße, 35033 Marburg, Germany; westhoff@med.uni-marburg.de (C.C.W.); brehmc@med.uni-marburg.de (C.U.K.); 3 Haematology, Oncology and Immunology, University Hospital Giessen and Marburg, Philipps University Marburg, Baldingerstraße, 35033 Marburg, Germany; trenker@med.uni-marburg.de; 4 Department Allgemeine Innere Medizin (DAIM), Kliniken Hirslanden Bern, Beau Site, Salem und Permanence, 3018 Bern, Switzerland; c.f.dietrich@googlemail.com; 5 Gastroenterology, Endocrinology, Metabolism and Clinical Infectiology, University Hospital Giessen and Marburg, Philipp University of Marburg, Baldingerstraße, 35033 Marburg, Germany; ajadalhyari@gmail.com

**Keywords:** peripheral organizing pneumonia, ultrasound, CEUS, histopathological correlation, CD34

## Abstract

**Purpose:** To describe the perfusion patterns of peripheral organizing pneumonia (POP) by contrast-enhanced ultrasound (CEUS) and their correlation with vascularization patterns (VPs) represented by immunohistochemical CD34 endothelial staining. **Methods:** From October 2006 until December 2020, 38 consecutive patients with histologically confirmed POPs were standardized-examined by CEUS. The time to enhancement (TE; classified as an early pulmonary-arterial [PA] pattern of enhancement vs. delayed bronchial-arterial [BA] pattern of enhancement), the extent of enhancement (EE; classified as marked or reduced), the homogeneity of enhancement (HE; classified as homogeneous or inhomogeneous), and the decrease of enhancement (DE; classified as rapid washout [<120s] or late washout [≥120s]) were evaluated retrospectively. Furthermore, tissue samples from the study patients were immunohistochemically stained with CD34 antibody. The presence of avascular areas (AAs) and the VPs were evaluated in all tissue samples. **Results:** The majority of POPs showed a BA pattern of enhancement (71.1%), an isoechoic marked enhancement (76.3%), and an inhomogeneous enhancement (81.6%). A rapid DE was observed in 50.0% of cases. On CD34 staining, all POPs had a chaotic VP, indicating BA neoangiogenesis. AAs (abscess, necrosis, hemorrhage) were identified in (41.9%) cases with an inhomogeneous enhancement on CEUS. **Conclusion:** On CEUS, POPs predominantly revealed a marked inhomogeneous BA pattern of enhancement with a rapid washout in 50% of cases. Furthermore, we demonstrated that the presence of a PA pattern of enhancement, found in 28.9% of POPs, did not exclude a BA neoangiogenesis as an important feature of chronic inflammatory and malignant processes.

## 1. Introduction

In 1901, the German pathologist W. Lange described for the first time the clinical and pathological features of organizing pneumonia (OP) on the basis of postmortem histological findings in two patients with cough, fever and dyspnea who died in hospital [1]. He termed the disease “bronchitis et bronchiditis obliterans” [1,2]. This term has since been replaced by the term “organizing pneumonia” to avoid confusion with the disease constrictive bronchiolitis obliterans [1,3]. OP is classified into cryptogenic and secondary OP. Cryptogenic OP cannot be attributed to a specific cause or a clinical constellation, whereas secondary forms of OP can be attributed to specific causes or may arise within a specific clinical context [1]. OP is a rare disease. A study from Iceland reported an incidence of 1.10/100,000 population for cryptogenic OPs and 0.87/100,000 for secondary forms of OP [4]. The radiologic findings on computed tomography (CT) or positron emission tomography–computed tomography (PET-CT) are nonspecific [1,5,6]. B-mode lung ultrasound (LUS) is considered to be a noninvasive complementary diagnostic method in addition to CT and PET-CT for the assessment of peripheral-based pulmonary lesions [7]. Furthermore, the visualization of the perfusion patterns of peripheral pulmonary lesions using contrast enhanced ultrasound (CEUS) improves the specificity of the B-mode LUS for the evaluation of these lesions and has been described for pleurisy, community-acquired pneumonia, pulmonary infarcts, peripheral granulomatous lesions, and peripheral lung cancer [8,9,10,11,12,13,14,15,16,17,18,19,20,21]. In contrast to the previous entities, to the best of our knowledge, neither B-mode LUS nor CEUS data for histologically confirmed OP have been reported in a large patient cohort. Therefore, knowledge regarding the features of these lesions in ultrasound as a widely used method in clinical practice is limited.

The aim of the present study was to describe the B-mode LUS features and the perfusion patterns of histologically confirmed peripheral organizing pneumonia (POP) using CEUS in a relatively large patient cohort and to correlate the sonographic findings with histopathological features and vascular patterns represented by immunohistochemical (CD34) endothelial staining in the corresponding lesions.

## 2. Materials and Methods

Between October 2006 and December 2020, a total of 598 patients with peripheral pulmonary lesions (PPLs) were examined prospectively, and were standardized using CEUS by a single German Society for Ultrasound in Medicine (DEGUM) Level-III qualified examiner with more than 35 years of experience in the field of thoracic sonography (C.G., internal medicine) at a university US center [22]. All the PPLs were over 5 mm in size and detected through conventional six-point B-mode LUS [23]. All the patients were referred to the US center for the investigation of a PPL and/or other thoracic pathologies. The inclusion criteria for the retrospective analysis were (1) histological confirmation of OP and (2) a time difference of ≤4 weeks between the CEUS examination and histopathological sampling. Ultimately, 38 patients with proven POP met the inclusion criteria and were included in the study. All the tissue samples from the study patients were immunohistochemically stained with CD34 antibody.

The ultrasound data were obtained according to the hospital guidelines during general clinical procedures, and they were prospectively collected and retrospectively evaluated. Informed consent was obtained from all the patients for the CEUS examination, and the study was approved by the local ethics committee and performed in accordance with the revised Helsinki Declaration.

### 2.1. Ultrasound Examinations

The B-mode LUS examinations were performed with an ACUSON SEQUOIA 512 GI ultrasound machine (Siemens, Erlangen, Germany) and a 4C1 curved-array transducer with a frequency of 4 MHz. The CEUS investigations were conducted with the same transducer in contrast-specific mode (1.5 MHz) and in accordance with the European Federation of Societies for Ultrasound in Medicine and Biology (EFSUMB) guidelines [24]. A bolus injection of 2.4 mL of the contrast medium SonoVue^®^ (Bracco Imaging S.p.A., Milan, Italy) was performed via peripheral venous access. This was followed by 10 mL NaCl 0.9%. For the first 30 s, the perfusion patterns of the lesions were continuously examined and recorded by a clip. Subsequently, several short examinations were performed at one-minute intervals up to 3 min, and the changes in the perfusion pattern were saved as images [17]. All ultrasound examinations were performed in the upright sitting position and horizontal to the ribs [17]. 

The B-mode LUS and CEUS data were evaluated retrospectively by two independent, experienced investigators (E.S., C.G.). In the event of discrepancies, the final decision was made by a third experienced investigator (C.T.). Cohen’s kappa statistics were applied to measure interrater reliability. The following B-mode LUS data and CEUS parameters were evaluated retrospectively [17]. 

### 2.2. B-Mode Lung Ultrasound Parameters

1. The echogenicity of the lesion was classified as hypoechoic or iso-/hyperechoic, compared with the echogenicity of parenchymal organs used as an in vivo reference [17].
2. The border of the lesion was classified as smooth or irregular-delineated [17].
3. The size of the peripheral pulmonary lesion was classified as having a ≥2 cm or <2 cm diameter [17].

### 2.3. Contrast-Enhanced Ultrasound Parameters

1. The time to enhancement (TE) of the contrast agent after intravenous injection was determined and classified as an early pulmonary-arterial (PA) pattern of enhancement (contrast enhancement of the lesion before the arrival of contrast agent in the thoracic wall) vs. delayed bronchial-arterial (BA) pattern of enhancement (contrast enhancement of the lesion simultaneous with the arrival of contrast agent in the thoracic wall or parenchymal organs) [17,19,25].
2. The extent of enhancement (EE) in the arterial phase was categorized as reduced EE (echo-free/hypoechoic) vs. marked EE (isoechoic) [17,19,25].
3. The homogeneity of enhancement (HE) was classified in the arterial phase as homogeneous enhancement vs. inhomogeneous enhancement of the POPs [14,16,17,18,20,26,27,28]. A perfused lesion with coexisting nonperfused areas (NPAs) was defined as an inhomogeneous enhancement [10,12,13,21].
4. The decrease of enhancement (DE) in the parenchymal phase (washout) was classified as a rapid washout (<120s) or a late washout (≥120s) [17,29].

The arterial phase was defined as the time from the earliest arrival of the contrast agent at the lesion to the peak of contrast agent enhancement of the lesion [17]. The parenchymal phase was defined as the time from the peak of contrast agent enhancement of the lesion to washout of contrast agent from the lesion [17]. Splenic tissue was considered as an in vivo reference to evaluate EE, HE, and DE (washout) of the contrast agent [17,19,30].

### 2.4. Histopathological Examination

**Figure 1:**
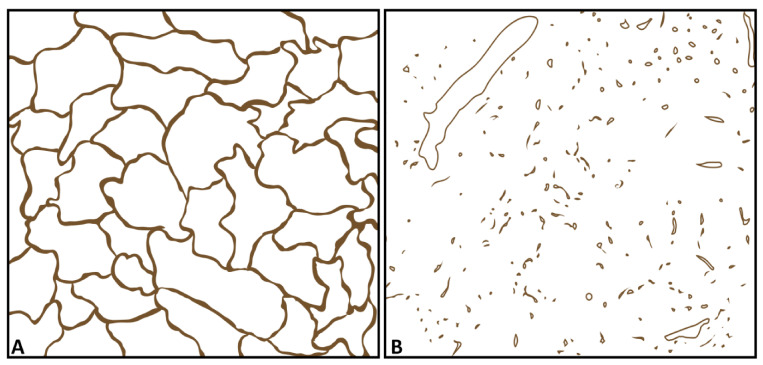
Graphical representation of patterns of immunohistochemistry for CD34 as a marker of endothelial cells [31]. (**A**) A regular alveolar pattern corresponding to the pulmonary capillary vascular pattern in healthy lung tissue [17] or acute pneumonia [17], as the corresponding pattern for pulmonary arterial supply. (**B**) A disorganized and chaotic vascular pattern similar to bronchial arterial neoangiogenesis in malignant lung tumors, as the corresponding pattern for bronchial arterial supply [17].

All tissue samples were fixed in 4% formalin solution, embedded in paraffin, cut at a thickness of 4 μm, and stained with hematoxylin and eosin (H&E) for routine purposes. Immunohistochemistry for CD34, a marker of endothelial cells [31], was performed using standard methods (EnVision+ Dual Link System-HRP, with 3,3′-diaminobenzidine as chromogen), detected by the monoclonal antibody QBEnd10 (each Agilent Dako, Waldbronn, Germany). All tissue samples were identified microscopically by an experienced pathologist (C.C.W.) as diseased lung tissue with OP. The following histopathological data were analyzed:

1. The presence of avascular areas in the POPs was determined in all lesions [17].
2. The vascular patterns with a regular alveolar pattern corresponding to the pulmonary capillary vascular pattern in healthy lung tissue [17] or acute pneumonia [17] as the corresponding pattern for PA supply (pattern A, Figure 1A), or disorganized and chaotic vascular patterns similar to BA neoangiogenesis in malignant lung tumors as the corresponding pattern for BA supply (pattern B, Figure 1B), were identified as described previously [17].

## 3. Results

### 3.1. Characteristics of the Participants

Of the 38 study patients, 31 were male and seven were female. The mean age of the patients was 60.8 ± 13.1 years (range 28–82 years). In 30 cases (78.9%) the diagnosis of secondary POP was made, and in eight cases (21.1%) the diagnosis of cryptogenic POP was made. Table 1 presents the final clinical diagnoses of POP in all study patients.

### 3.2. B-Mode Lung Ultrasound Data

**Figure 2:**
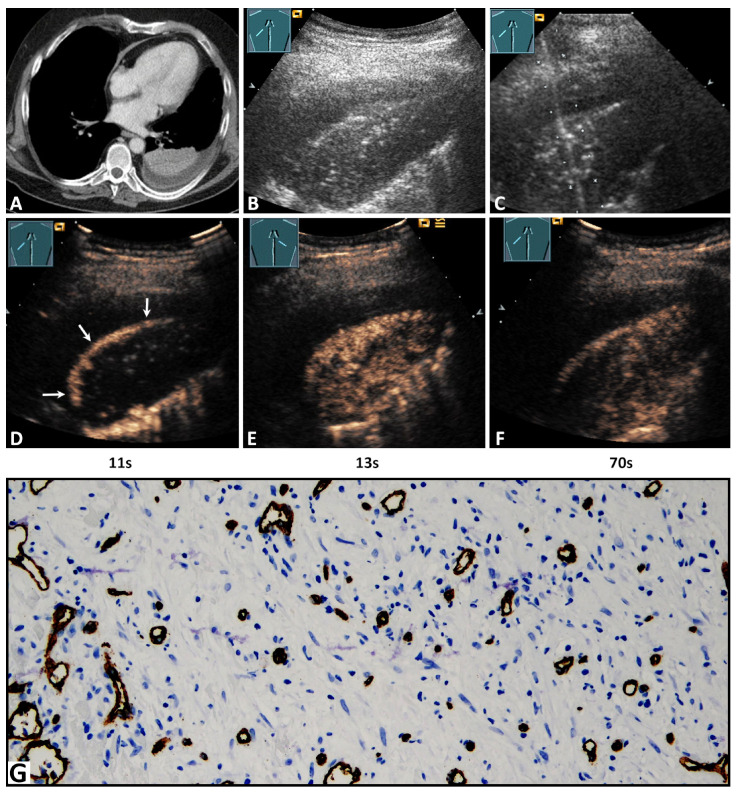
A 62-year-old male patient with lung consolidation on (**A**) computed tomography (courtesy of Prof. Dr. Andreas H. Mahnken, Department of Radiology, University Hospital Marburg) and (**B**) B-mode ultrasound. (**C**) An ultrasound-guided 18G-core needle biopsy of the lung lesion was performed. The histopathological examination of the lesion presented the diagnosis of organizing pneumonia. On contrast-enhanced ultrasound, the lesion showed a delayed enhancement due to (**D**) a bronchial-arterial supply by peripheral (arrows) bronchial arteries, and (**E**) a hypoechoic and inhomogeneous pattern of enhancement with (**F**) an early decrease of enhancement. In the tissue sample, (**G**) immunohistochemical staining with CD34 was performed, and reorganized lung tissue with fibrotic tissue and a high capillary density in a rather disorganized and chaotic pattern was present, consistent with pattern B (×200).

**Figure 3:**
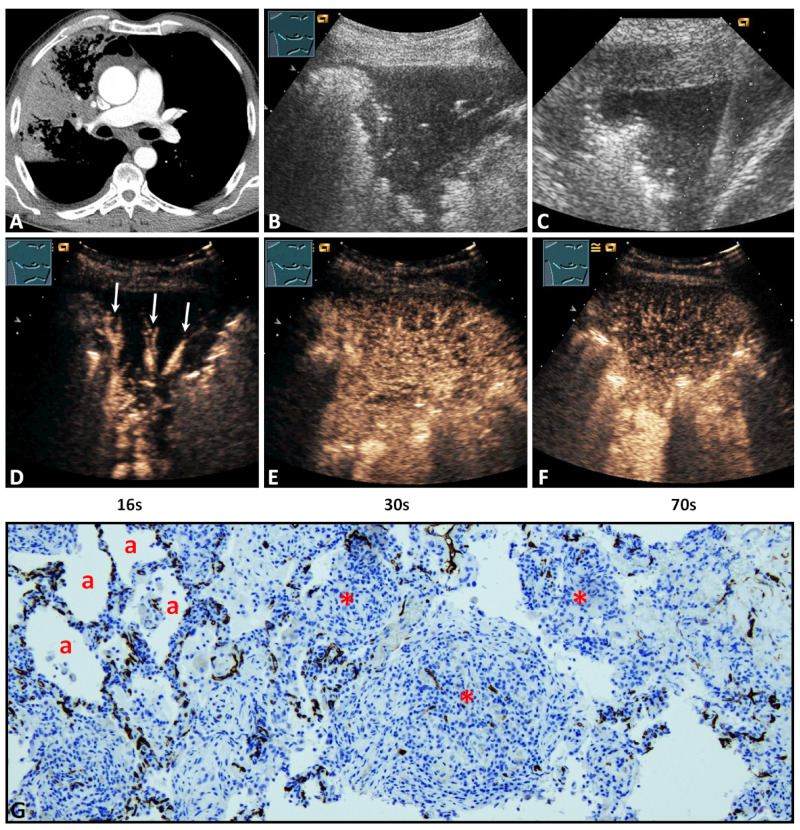
A 59-year-old male patient with lung consolidation on (**A**) computed tomography (courtesy of Prof. Dr. Andreas H. Mahnken, Department of Radiology, University Hospital Marburg) and (**B**) B-mode ultrasound. (**C**) An ultrasound-guided 18G-core needle biopsy of the lung lesion was performed. The histopathological examination of the lesion presents the diagnosis of organizing pneumonia. On contrast-enhanced ultrasound, the lesion showed (**D**) a pulmonary-arterial (arrows), (**E**) isoechoic, and homogenous pattern of enhancement (**F**) with an early decrease of enhancement. (**G**) In the tissue sample, immunohistochemical staining with CD34 was performed. To the left, preserved alveolar lung vasculature surrounding normal or macrophage-filled lung alveoli (a) is evident in favor of pattern A. On the right, reorganized lung tissue with fibrotic and granulating tissue with a high capillary density in a rather disorganized and chaotic pattern (*) is present, consistent with pattern B (×100).

On B-mode LUS, all POPs were hypoechoic (Figure 2B and Figure 3B). In 21/38 cases (55.3%) POPs showed an irregular border, and in 17/38 cases (44.7%) they showed a smooth delineated border. The size of the POPs was ≥2 cm in 34/38 (89.5%) cases and <2 cm in 4/38 cases (10.5%).

### 3.3. Contrast-Enhanced Ultrasound Data

Regarding TE, 27/38 lesions (71.1%) revealed a delayed enhancement due to BA perfusion (Figure 2D), and 11/38 lesions (28.9%) revealed an early enhancement due to PA perfusion (Figure 3D). Regarding EE, 29/38 cases (76.3%) revealed a marked enhancement (Figure 3E) and 9/38 cases (23.7%) a reduced enhancement (Figure 2E). Furthermore, 7/38 lesions (28.4%) showed a homogeneous arterial enhancement (Figure 3E), and 31/38 lesions (81.6%) showed an inhomogeneous arterial enhancement with evidence of NPAs on CEUS (Figure 2E). The DE (washout time) was rapid (<120 s) in 19/38 lesions (50.0%) and late (≥120 s) in 19/38 lesions (50.0%) (Figure 2F and Figure 3F). 

The agreement between the examiners for the ultrasound finding was “very good” (Cohen’s kappa = 0.70).

### 3.4. Histopathological Data and Their Correlation with Contrast-Enhanced Ultrasound Pattern

All POPs were histologically confirmed to exclude malignancy. The histological confirmation was performed by ultrasound-guided biopsy in 25 cases (65.8%) (Figure 2C and Figure 3C) and by a thoracic surgical intervention in 13 cases (34.2%). The mean time difference between CEUS and histopathological sampling was 1.8 ± 1 weeks (range 1–4 weeks). A vascular pattern B similar to BA neoangiogenesis in lung tumors was found in all lesions (Figure 2G and Figure 3G). Moreover, in the 11/38 lung lesions (28.9%) that had a PA pattern of enhancement, the vascular pattern A similar to PA supply in healthy lung tissue [13] or acute pneumonia [13] was identified in addition to vascular pattern B (Figure 3G).

On immunohistochemical staining with CD34 antibody, avascular areas (abscess, necrosis, hemorrhage) in POP were identified in 13/31 cases (41.9%) with an inhomogeneous enhancement on CEUS.

## 4. Discussion

The diagnosis of OP is made according to defined histopathological criteria [1]. In the case of a histological diagnosis of OP, malignant or systemic inflammatory disease should be excluded, because in most patients an underlying disease is found as the cause of the OP [1,32]. In this study, the cause associated with OP was identified in 78.9% of patients, and these cases were classified as secondary OP. If the cause is not found, a careful follow-up should be performed in these patients in addition to the corresponding therapy [32]. If the underlying cause is not found in the follow-up, the disease is clinically termed as cryptogenic OP. The decision for histological confirmation as the first step in the diagnosis of POP is based on the clinical presentation and clinical background of the patient, as well as on radiological features of the lesions. In a standardized study over the course of 14 years, we studied such features of POP in CEUS.

To interpret the perfusion patterns of different pathologies using CEUS, it is essential to understand the corresponding histopathological vascularization features of the underlying lesion [17]. A recently performed study in granulomatous lung lesions demonstrated that a disorganized and chaotic vascular pattern on immunohistochemical staining with CD34 antibody could be considered as neoangiogenesis by bronchial arteries and could be correlated to a BA pattern of enhancement on CEUS regarding TE, EE, and DE [17]. Furthermore, in acute pneumonia, which is predominantly associated with a PA pattern of enhancement, and in normal lung tissue, the corresponding histopathological correlation [17] showed a regular alveolar vascular pattern on immunohistochemical staining with CD34 antibody [17]. 

Regarding TE in this study, on CEUS, POPs had a predominantly (71.1%) delayed systemic enhancement in the arterial phase, indicating a BA supply. In all tissue samples, a disorganized and chaotic vascular pattern was seen (Figure 2) as evidence of BA neoangiogenesis [17,33]. Furthermore, in all lesions with a PA perfusion pattern of enhancement (28.9%), a regular alveolar vascular pattern was partially present in the corresponding tissue sample as an indication of PA supply (Figure 3) [13]. These findings indicate that, in the presence of a PA pattern of enhancement, a BA neoangiogenesis, as an important feature of chronic inflammatory and malignant processes, cannot be excluded by CEUS. In these lesions, early PA enhancement covered the delayed arrival of contrast medium via BA supply. 

Regarding HE and EE, 81.6% of cases presented an inhomogeneous enhancement and 76.3% a marked arterial enhancement in comparison with splenic enhancement. Avascular areas (abscess, necrosis, hemorrhage), as a histopathological correlation to an inhomogeneous enhancement, were identified in 13/31 cases (41.9%). In the remaining cases, a histopathological correlation was not demonstrated, based on the fact that avital tissues were avoided in the biopsy or histopathological examination. All POPs with a PA pattern of enhancement showed a marked enhancement similar to other pathologies with PA supply, such as compression atelectasis and acute pneumonias [9,13,19]. Furthermore, 18/27 lesions (66.7%) with a BA pattern of enhancement showed an isoenhancement that may be indicative of a marked neoangiogenesis [17]. 

Regarding DE, 50% of POPs revealed a rapid decrease of enhancement (<120s) in the parenchymal phase. Furthermore, it was demonstrated that the presence of late DE (>120s), as a further feature of benign pulmonary lesions [29], did not also exclude a BA neoangiogenesis.

Depending on the histopathologic features, peripheral pulmonary lesions may present similar perfusion patterns in CEUS regarding TE, EE, HE, and DE (Table 2). In this context, the chronic processes predominantly demonstrate a BA pattern of enhancement due to BA neoangiogenesis (Table 2). Furthermore, the chronic processes may reveal a rapid DE due to the abnormal veins and arteriovenous shunts associated with neoangiogenesis [17,29,34].

There are some limitations to this study. These include the retrospective nature of the investigation and the general limitations of ultrasound examinations, which are characterized by a high interobserver and interequipment variability. Furthermore, only approximately 70% of the pleural surface can be visualized in a US examination. Due to physical phenomena, such as interfacial reflections by air (with resulting artefacts) and absorption on bony structures, LUS is limited to pleural-based lesions located in visible parts of the visceral pleura [37,38,39]. It was not possible to blind the investigators to the study group, and blinded interpretations of the ultrasound data by the ultrasound examiner were not possible. Moreover, POPs are relatively rare; all the patients included in the study were referred to the Interdisciplinary Centre of Ultrasound Diagnostics for the investigation of a PPL and/or other thoracic pathologies, and were investigated in a standardized way by a single DEGUM-qualified Level-III examiner using the same B-mode LUS and CEUS protocol. Therefore, the data collection was conducted over a long period of time, and selection bias cannot be excluded. Moreover, the semi-quantitative classification of the findings probably leaves more room for interpretation than a quantitative measurement, whereby the interrater-observer variability for the ultrasound finding was made with “very good” agreement.

## 5. Conclusions

In summary, we found that POPs show a nonspecific pattern on CEUS. Predominantly, a marked inhomogeneous BA pattern of enhancement was seen, and there was a rapid (<120s) DE (washout) in 50% of cases. Furthermore, we demonstrated that the presence of a PA pattern of enhancement or delayed washout could not exclude a BA neoangiogenesis as an important feature of chronic inflammatory and malignant processes. In these lesions, the underlying BA supply is covered by the early PA pattern of enhancement. Because of an overlap of CEUS patterns between chronic inflammatory and malignant peripheral pulmonary lesions, the histological examination of these lesions is warranted.

## Figures and Tables

**Table 1:** Final clinical diagnoses of *N* = 38 study patients with peripheral organizing pneumonia (POP).

POP Classification	*n*/*N* (%)
Cryptogenic POP	8/38 (21.1%)
Secondary POP - Infections - Nonhematologic malignancies - Hematologic malignancies - Systemic inflammatory diseases	30/38 (78.9%) 11/38 (28.9%) 9/38 (23.7%) 5/38 (13.2%) 5/38 (13.2%)

POP: peripheral organizing pneumonia.

**Table 2:** Contrast-enhanced ultrasound perfusion patterns of pulmonary inflammatory and neoplastic lesions.

Underlying Disease	Acute Pneumonia	Neoplastic Pulmonary Lesions	Granulomatous Disease	Organized Pneumonia
Author	Linde et al. [35]	Sartori et al. [36] *	Safai Zadeh et al. [17]	Present study
Cases	50	53	10	38
Year	2012	2013	2021	2021
Pattern of enhancement on CEUS				
TE: PA	92.0%	5.7%	0%	28.9%
BA	8%	94.3%	100%	71.1%
EE: Isoechoic	74%	54.7%	0%	76.3%
Hypoechoic	26%	45.3%	100%	23.7%
HE: Hom	78%	11.3%	0%	18.4%
Inhom	22%	88.7%	100%	81.6%
DE: Rapid	Not analyzed	98.1% *	100%	50%
Late		1.9% *	0%	50%

CEUS: contrast enhanced ultrasound; PA: pulmonal arterial; BA: bronchial arterial; Hom: homogeneous; Inhom: inhomogeneous; TE: time to enhancement; EE: extent of enhancement; HE: homogeneity of enhancement; DE: decrease of enhancement. * In this study, decrease of enhancement was in 98.1% < 180s and in 1.9% > 180s.

## Data Availability

The data presented in this study are available on request from the corresponding author.
